# Coffee Consumption and C-Reactive Protein Levels: A Systematic Review and Meta-Analysis

**DOI:** 10.3390/nu12051349

**Published:** 2020-05-08

**Authors:** Elizabeth D. Moua, Chenxiao Hu, Nicole Day, Norman G. Hord, Yumie Takata

**Affiliations:** 1 College of Pharmacy, Oregon State University/Oregon Health & Science University, Corvallis, OR 97331, USA; mouae@oregonstate.edu; 2 Department of Statistics, College of Science, Oregon State University, Corvallis, OR 97331, USA; huche@oregonstate.edu; 3 College of Engineering, School of Chemical, Biological, and Environmental Engineering, Oregon State University, Corvallis, OR 97331, USA; Nicole.B.Day@colorado.edu; 4 College of Public Health and Human Sciences, School of Biological and Population Health Sciences, Oregon State University, Corvallis, OR 97331, USA; norman.hord@oregonstate.edu

**Keywords:** coffee consumption, c-reactive protein, cross-sectional studies, systematic review and meta-analysis

## Abstract

Coffee contains bioactive compounds with anti-inflammatory properties, and its consumption may reduce c-reactive protein (CRP) levels, a biomarker of chronic inflammation. A previous meta-analysis reported no overall association between blood CRP level and coffee consumption by modeling the coffee consumption in categories, with substantial heterogeneity. However, the coffee cup volume was not considered. We conducted a systematic review and dose–response meta-analysis investigating the association between coffee consumption and CRP levels reported in previous observational studies. A dose–response meta-analysis was conducted by mixed-effects meta-regression models using the volume of coffee consumed as metric. Eleven studies from three continents were identified using the PubMed database, totaling 61,047 participants. Three studies with the largest sample sizes observed a statistically significant association between coffee and CRP levels, which was inverse among European and United States (US) women and Japanese men (1.3–5.5% decrease in CRP per 100 mL of coffee consumed) and positive among European men (2.2% increase). Other studies showed no statistically significant associations. When all studies were combined in the dose–response meta-analysis, no statistically significant associations were observed among all participants or when stratified by gender or geographic location, reflecting the conflicting associations reported in the included studies. Further studies are warranted to explore these inconsistent associations.

## 1. Introduction

Coffee is a well-known beverage around the world and the most popular caffeinated drink choice [1,2]. A recent meta-analysis of 31 cohort studies reported that coffee consumption is associated with decreased risk of total mortality and cause-specific mortality from cardiovascular disease (CVD) and cancer [3]. C-reactive protein (CRP) is considered a biomarker of chronic inflammation [4] and of disease risk and progression [5], including for CVD. The association between coffee and mortality reported in the meta-analysis may be mediated through CRP. 

Coffee contains many bioactive components such as chlorogenic acids, polyphenols, diterpenes, micronutrients and caffeine [2,6,7,8], which may exert beneficial health effects through antioxidant and anti-inflammatory properties [6,7,8]. There are also bioactive compounds that may negatively affect health; for instance, the high amount of lipids in unfiltered coffee may increase blood cholesterol [9,10].

In this analysis, we investigated the associations between coffee consumption and CRP levels among adults in observational studies by conducting a systematic literature review and a dose-response meta-analysis. A previous meta-analysis of 24,863 participants from nine studies modeled coffee consumption by categories and reported no overall statistically significant association with CRP level, but there was evidence of substantial heterogeneity [11]. The cup volume varied by geographic location, which affects the amount of bioactive compounds consumed and, consequently, their biological effects [7,8,12,13,14]. Hence, we modeled the volume of coffee consumed and hypothesized that higher coffee consumption is associated with lower levels of CRP.

## 2. Materials and Methods

### 2.1. Registration

This study was reported according to the Preferred Reporting Items for Systematic Reviews and Meta-Analyses (PRISMA) [15] and is registered with the PROSPERO International Prospective Register of Systematic Reviews (CRD42018108351). 

### 2.2. Literature Search

A systematic literature review was conducted using the electronic database PubMed to collect data from published studies. The following search terms were used: (“coffee” [MeSH Terms] OR “coffee”[All Fields]) AND (“c-reactive protein”[MeSH Terms] OR (“c-reactive”[All Fields] AND “protein”[All Fields]) OR “c-reactive protein”[All Fields] OR “c reactive protein”[All Fields]). The last search was conducted in March 2020, and a total of 61 abstracts were identified and reviewed independently by two authors (E.D.M. and Y.T.). A full-text article was not obtained if the title and/or abstract met one or more of the following exclusion criteria: (1) animal study; (2) study among children; (3) no mention of coffee or other beverages, or of associations between food or beverage intake other than coffee and CRP or other inflammatory biomarkers; (4) no mention of CRP or other inflammatory markers/biomarkers, or of associations between beverage consumption and outcome variables other than CRP; (5) non-original study such as review or commentary. After removing abstracts that met those exclusions, the full-text articles of the remaining abstracts were obtained, reviewed, and excluded if they met one of the following additional exclusion criteria: (1) not reporting the association between coffee consumption and CRP level; (2) overlapping study populations; (3) not reporting enough data, such as coffee consumption categories (at least three) and levels of CRP within those categories; (4) meeting any of the previous exclusion criteria that could not be determined from the abstract alone, but was determined based on the full-text article. A total of 10 articles that did not meet any of the exclusion criteria were included in the systematic literature review. We found three meta-analysis/systematic review articles in our PubMed search [4,11,16] and compared the list of original research studies included in the article, finding no additional studies to the 10 articles we identified that met our inclusion criteria. Data were extracted independently by two authors (E.D.M. and Y.T.) and inconsistencies were discussed and brought to consensus.

The following information was extracted, compiled and summarized for each study: first author; year of publication; study name; country; study design; calendar years when the study was conducted and questionnaire information and blood samples were collected; number of participants; age; gender; coffee consumption assessment (questionnaire validated or not) and methods of questionnaire administration (interview or self-administered); cup volume conversion and/or frequency of consumption; study results such as CRP levels (mean and standard deviations/error or 95% confidence intervals (CI)) by coffee consumption categories. 

Not all of the information described above could be extracted from each of the 10 articles. In such cases, articles that might provide missing information were examined by examining references cited by the studies, searching for articles about the study or questionnaire through PubMed, or contacting the corresponding author of the articles. For instance, information on cup volume conversion was available for nine studies [17,18,19,20,21,22,23,24], but not for the Kyushu University cohort and the Dose-Response to Exercise in Women (DREW) trial [25,26]. When we contacted the corresponding authors of these two studies, we either received confirmation of the lack of information [26] or no response [25]; hence, we estimated the cup conversion based on the other studies conducted in similar location and calendar years, such as the Aichi Workers’ study [20] and Nurses’ Health Study (NHS) [18], respectively. Data on high-sensitivity CRP levels were obtained from all studies, but two studies needed to be converted to milligram per liter [20,22]. For all 11 studies, high-sensitivity CRP was measured using blood samples collected throughout each study. 

The risk of bias for each study was assessed by both authors (E.D.M. and Y.T.) independently using the modified Newcastle–Ottawa Scale for cross-sectional studies [27]; any discrepancies were discussed and a consensus reached.

### 2.3. Meta-Analysis

A meta-analysis of associations between coffee consumption volume and CRP level was conducted through the Metafor package from R. Based on the cup conversion information we collected and the range of cups of coffee consumed in each category reported in each article, we estimated the mid-point volume (in mL) of coffee consumption in each category; we then re-calculated the *p*-trend to test a linear association between coffee consumption and CRP level by treating coffee volume as continuous in a model.

The estimated weighted mean changes in CRP level (per 100 mL of coffee) and 95% CI were calculated through a mixed-effects meta-regression model. We used log-transformed CRP levels in our meta-analysis. Due to incomparability of the reported values, the ATTICA study was excluded from the meta-analysis. The heterogeneity across studies was assessed by *I*^2^ statistics [28]. To explore the source of heterogeneity, we pre-specified and stratified analyses by gender and geographic location.

## 3. Results

**Figure 1:**
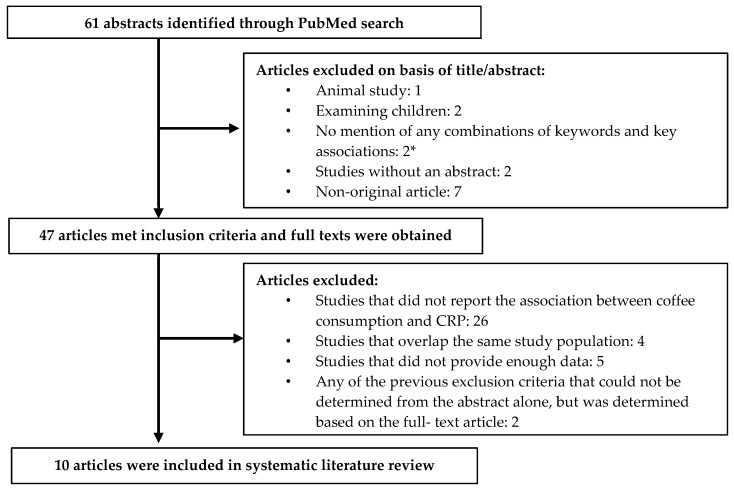
Flow chart of systematic literature review. * no mention of coffee or other beverages, or of associations between food or beverage intake other than coffee and c-reactive protein (CRP) or other inflammatory biomarkers; no mention of CRP or other inflammatory markers/biomarkers, or of associations between beverage consumption and outcome variables other than CRP.

Our study identified 61 abstracts published in March 2020 or earlier with search terms identified previously. After reviewing articles based on the title and abstract (Figure 1), 47 were selected to obtain full-text articles. Among them, 37 of the published studies were excluded: 26 for not reporting the association between coffee consumption and CRP level, four for using the same study population [29,30,31,32], five for not providing enough data [33,34,35,36,37], and two for being a non-original article [38,39]. In total, 10 published articles reporting associations between coffee consumption and CRP levels in 11 study populations (one article reported results from two cohorts [18]) were selected and included in the current systematic review and meta-analysis. 

The 11 studies were conducted between 1976 and 2007 in various locations: three in the United States (US) [18,26], three in Asia [19,20,25], and the rest in Europe [17,21,22,23,24] (Table 1). These 11 studies involved a total of 61,047 participants. Most studies had over 500 participants each [17,18,19,20,22,23,25], with the exception of two studies which had 344 and 61 participants, respectively [21,26]. Among the 11 studies, six included both men and women [17,19,20,22,23,25]. Of the other five studies, two included only women [18,26] and three included only men [18,21,24]. 

The age of study participants varied from 18 to 87 years old. The mean body mass index (BMI) of each study was between 22.8 kg/m^2^ and 36.1 kg/m^2^, with the exception of one that did not report BMI values, but reported instead that 17.7% of participants were obese [17]. Six studies reported that the mean alcohol consumption ranged from 1.7–27.3 g/day [18,20,21,22,23,24,26], while four studies reported that 18.6–72.7% of participants drank alcohol [19,25]. One study did not provide information on alcohol consumption [17]. As for smoking, one study of women and one study of men consisted of only non-smokers [21,26]. Hence, the percentage of current smokers ranged from 0% (due to recruitment criterion) in the DREW trial and United Kingdom (UK) study [21,26] to 53.3% in the ATTICA study [17]. Of the three studies that included postmenopausal women and reported hormone therapy use [18,22,26], the proportion of users ranged from 25.9% in the European Prospective Investigation into Cancer and Nutrition (EPIC) study [22] to 46.5% in the DREW trial [26].

All studies used questionnaires to assess coffee consumption, through self-administration, interview, or both. Each study had 3–5 coffee consumption categories including “no”, “low”, “medium”, “high”, and “very high” coffee consumption. The consumption amount considered “low” or “high” varied across studies, ranging from 1 cup/month to <6 cups/week for low consumption and 2 to ≥6 cups/day for high consumption. Two studies included the fifth (“very high”) consumption category as high as ≥7 cups/day [22,25]. As the studies were conducted in various locations, the volume of a cup for each region ranged from 150 mL in the Aichi Workers’ cohort in Japan [20] and the Finnish Diabetic Nephropathy (FinnDiane) study in Finland [23] to 237 mL in NHS and Health Professional Follow-Up Study (HPFS) studies in the US [18] and EPIC and UK studies in Europe [21,22]. 

When the linear association was assessed taking the volume of coffee consumed into account, three of the 11 studies observed statistically significant inverse or positive associations between coffee consumption and CRP levels, although the rest reported no significant association (Table 2). These three studies examined associations separately by gender, and different associations were observed by gender and study [18,22,25]. Among women, the EPIC and NHS included 10,520 and 15,551 women, respectively, and they had a statistically significant inverse association (EPIC: *p*-trend = 0.002, 2.7% decrease in CRP per 100 mL of coffee consumption; NHS: *p*-trend = 0.02, 5.5% decrease in CRP) [18,22]. Among men, the Kyushu University cohort included 4407 men and also showed a statistically significant inverse association (*p*-trend = 0.03, 1.3% decrease in CRP) [25]. In contrast, the EPIC study had 4280 men and showed a statistically significant positive association between coffee consumption and CRP levels (*p*-trend = 0.01, 2.2% increase in CRP) [22]. 

When the 10 studies that reported compatible CRP levels were combined through the dose–response meta-analysis, no statistically significant association was observed among all participants (mean change in CRP level: −2.4%; 95% CI: −8.7% to 4.4%; *p* = 0.49) with no evidence of heterogeneity (*I*^2^ < 0.01%). Similarly, no significant associations (mean change in CRP level; 95% CI; *p*-value) were observed when we stratified by gender (men: 0.9%; −9.7% to 8.8%; *p* = 0.85; women: −6.3%; −16.4% to 5.0%; *p* = 0.26), or geographic location (US: −6.7%; −6.5% to 8.3%; *p* = 0.54; Europe: 0.6%; −6.5% to 8.3%; *p* = 0.83; Asia: −1.6%; −12.1% to 10.2%; *p* = 0.78) with no evidence of heterogeneity (*I*^2^ < 0.01%). Given that BELSTRESS study [24] was based on crude estimates, we repeated the analyses without the BELSTRESS study, which did not materially change the results (data not shown).

Regarding risk of bias, assessed through the modified Newcastle–Ottawa Scale for cross-sectional studies [27], all studies had scores of six or higher (Supplementary Table S1). Five studies [20,21,22,23,25] scored seven, which is considered good quality. Two studies [24,26] scored six (satisfactory quality), three [18,19] scored eight (good quality), and another [17] scored 10 (very good quality).

## 4. Discussion

To our knowledge, this is the first dose–response meta-analysis of the association between coffee consumption and CRP level in cross-sectional analyses that considered the volume of coffee consumed instead of categorical data, as employed in a previous meta-analysis [11]. Hence, our analysis is more robust in assessing the effects of volume of coffee consumed, which reflects the amount of bioactive compounds in coffee better than considering categories alone [12,13,14]. This is important as cup volume varied across studies (150 to 237 mL). When studies were combined through the dose–response meta-analysis, no statistically significant associations were observed between coffee consumption and CRP levels among all studies or after stratifying by gender or geographic location. To further elucidate this finding, we examined associations of CRP level with coffee consumption by modeling the volume of coffee and re-calculating the *p*-value for linear associations for each study. We found that the three studies with the largest sample sizes, NHS, EPIC study, and Kyushu University cohort, had statistically significant inverse or positive associations between coffee consumption and CRP levels, while others reported no statistically significant association. These inconsistent associations across studies may be explained by differences in study populations as discussed below. 

The characteristics related to coffee preference and preparation methods common in each study population may have affected the associations between coffee consumption and CRP level due to variation in the amount of bioactive compounds [12,13,47,48]. Around the time when coffee consumption was assessed in the included countries, instant coffees were popular in all countries. Unfiltered coffee was more commonly consumed in European countries than in the United States, Japan, and Singapore, where filtered coffee was more common [49]. Among the 11 included studies, three investigated associations separately by coffee preparation methods or types—filtered and unfiltered coffees in the ATTICA study [17] and decaffeinated and caffeinated coffees in the NHS and HPFS [18]. However, all studies found similar associations for either method [17,18]. Moreover, previous coffee intervention trials reported no difference in CRP levels by different types or preparation methods of coffee [34,50,51]. Nevertheless, a previous animal study provided evidence of anti-inflammatory effects of caffeine, given that a three-week caffeine administration (7.5 to 15 mg/kg of body weight) resulted in decreased CRP level in rats [7]. Therefore, previous human studies [34,50,51] may not have had a sufficiently large variation in coffee consumption (due to small sample sizes or homogeneous coffee consumption within a single study population) to observe differences in CRP levels comparable to the animal study [7]. Our meta-analysis had a limited ability to further explore associations by coffee preparation methods or types given that only three of the included studies conducted separate analyses [17,18]. In addition to caffeine, chlorogenic acid was reported to decrease inflammation in vitro, and its content varies by roasting levels [52]. To provide greater insight, future human studies may need to assess type, preparation methods and roasting types, along with the volume of coffee consumed. 

In addition, discrepant results between studies could be due to participants’ characteristics common in groups defined by gender and geographic location. These characteristics might have contributed to their interactions with coffee consumption or have confounded the observed associations. Firstly, BMI may have affected the inconsistent results between European men and women and Japanese men. Given the pro-inflammatory nature of CRP, a positive association of CRP levels with BMI and body fat composition was reported previously [53]. The EPIC study, with a positive association, had a higher average BMI (26.3 kg/m^2^) [44] than the Kyushu University cohort (23.5 kg/m^2^) [25], which reported an inverse association. Similarly, within the same EPIC study population, opposing associations (a positive association for men and an inverse association for women) were reported, which suggests that BMI (26.3 kg/m^2^ for men and 24.2 kg/m^2^ for women on average) [44] might have played a role rather than other factors such as coffee type. Hence, it is possible that BMI or body fat mass may have contributed to conflicting associations of coffee consumption with CRP levels in the EPIC and Kyushu University cohort studies. To further elucidate potential involvement of body fat mass, future studies need to use other anthropometric measures that are more reflective of body fat mass than BMI and stratify results by these anthropometric factors. 

Secondly, smoking might have contributed to discrepant results. CRP levels were higher among smokers than non-smokers [54], and smoking tends to be more common among men than women, especially in Japan [25,55] and Singapore [56]. In addition, confounding effects of smoking in the association between coffee and CRP level are possible as previously reported for coronary artery disease or CVD mortality [3,57], which were closely linked to CRP levels. For the studies with statistically significant associations, the proportion of current smokers was 32% for men in the Kyushu University cohort [25], 30% for men and 17% for women in the EPIC study [45], and 13% in the NHS [18]. Hence, the relatively low proportion of current smokers might partially explain the inverse association for European and US women. However, other factors may play a role in conflicting associations between European and Japanese men, which warrants further investigations. 

Alcohol intake may have also been an influential variable in gender differences with regard to the association between coffee consumption and CRP level. Among women, studies with a significant inverse association tended to have a higher alcohol consumption (6.0 g/day in NHS [18] and 4.2 g/day in EPIC women [22]) than studies with a non-significant association (1.7 g/day in DREW trial [26] and 27.1% as alcohol consumers in women in Kyushu University Cohort [25]). Among men, however, this trend was not clearly observed; EPIC men with a significant positive association reported consumption of 14.5 g/day [22]; Kyushu University Cohort men with a significant inverse association reported 72.7% of current alcohol consumers [25]; other studies with non-significant associations reported consumption in the range of 11.8 g/day in HPFS [18] and 27.3 g/day in BELSTRESS [24]. Among men in the Kyushu University Cohort, the inverse association between coffee and CRP was strongest among high current alcohol consumers [25], which might have driven the overall inverse association when all men in this study were combined. In the EPIC study, the association between coffee and CRP was not reported by alcohol consumption level. Biological effects may be influenced by consumption differences between genders, suggesting that the inverse association may be stronger for drinkers than non-drinkers. The positive association observed in EPIC men [22] may be partially explained by a previously reported U-shaped association between alcohol consumption and CRP [58], which warrants further investigation. The stronger inverse association between coffee and CRP in high alcohol consumers among Japanese men [25] is not consistent with the reported U-shaped association; however, relatively lower BMI in Japanese men than European men might have contributed to this difference. Additionally, the proportion of smokers was similar between Japanese and European men, which may not explain the opposing associations reported. Taken together, other potential factors (e.g., types of alcoholic beverages) that may explain the discrepant associations between European and Japanese men need to be explored in future studies. 

Strengths of this current analysis include the large number of participants (a total of 61,047), with a majority of the included studies comprising over 500 participants. This analysis included a diverse population of apparently healthy men and women, overweight and postmenopausal women, and men and women with known diabetes or metabolic syndrome. Additionally, this analysis obtained results from over 15 different countries in Europe, North America, and Asia. This diversity in study populations allowed us to cover a wide range of coffee consumption levels (the estimated median volume of coffee consumed ranged from 150 mL in Aichi Workers’ cohort [20] to 570 mL in the FinnDiane study [23]) that could not be achieved within a single study, and it allowed us to conduct a thorough examination of the association between coffee consumption and CRP levels. We also estimated the volume of coffee consumed in our analysis, instead of the pre-defined category or number of cups consumed, which better reflects the amount of hypothesized bioactive compounds in coffee. In addition, the comparability of CRP values across studies is a strength as all studies measured high-sensitivity CRP. The duration of blood sample storage varied by study; however, CRP values were reported to be highly stable over time (spanning several years) when blood samples were stored under the well-kept conditions [59] that all included studies followed. There may be slight variations in blood collection and handling procedures (such as temperature or time between blood collection and storage), which were also reported not to affect CRP values [60].

A limitation of this analysis is that the sample size ranged from 61 to 15,551, although a majority of the studies had over 500 participants. This may have affected the number of coffee consumption categories and statistical power within a single study. Hence, we conducted a dose–response meta-analysis including all eligible studies. Secondly, regarding CRP levels, two studies of women in the United States (i.e., DREW trial and NHS) had relatively higher levels than the rest of the studies. Previously, obesity and hormone therapy use were reported as determinants of high CRP level [38,53,61]. Both studies [18,26] had the two highest proportions of hormone therapy users (46.5% and 41.3% for the DREW trial and NHS, respectively), and the DREW trial included only overweight and obese women, while the NHS had a higher average BMI than other studies; these two characteristics may explain their high CRP levels. Thirdly, except for sex, we had limited ability to explore effects of potential confounding factors such as smoking and BMI. This is due to the fact that we did not have participant-level data and we relied on the reported associations adjusted for a set of confounding variables chosen by study investigators. Moreover, our study was limited in exploring the confounding effect of age because age ranges of study participants overlapped among the included studies and no study conducted analyses stratified by age group. Fourthly, all studies were based on cross-sectional analyses, which cannot infer temporal association, and future prospective analyses are warranted. Fifthly, our analysis only included one biomarker of chronic inflammation, CRP. Thus, other biomarkers such as interleukin-6 and tumor necrosis factor-alpha need to be explored in future studies. These biomarkers were linked to coffee extracts in previous in vitro [52] and human studies [17,18]. Furthermore, there is recent development in isoforms of CRP, such as pentameric and monomeric isoforms, linked to cardiovascular diseases and inflammatory conditions [62], which need to be considered in future studies. Sixthly, all the included studies used a food frequency questionnaire (FFQ) to assess coffee consumption. Although most were previously validated and reported correlation coefficients of coffee intake between FFQ and other dietary assessment instruments as high as 0.78 [31], the use of FFQs may have impacted the coffee consumption data due to inherent self-reporting errors [63]. Nonetheless, it is an efficient way to assess dietary intake in a large sample size. Future studies could use biomarkers of coffee consumption (e.g., urinary furoylglycine [64,65], *N*-methylpyridinium, and trigonelline [66]) to overcome limitations in self-reporting methods. Additionally, these biomarker studies would address the potential difference in the amount of bioactive compounds resulting from differences in coffee preparation, brew strength, roasting and beans, which cannot be construed solely by the volume of coffee consumed. Future studies measuring biomarkers of coffee consumption or collecting detailed information on coffee type and preparation method are warranted. 

## 5. Conclusions

Our results from the dose–response meta-analysis suggest no statistically significant association between coffee consumption and CRP level among all studies combined or after stratification by gender and geographic location. The three individual studies with the largest sample sizes among the 11 included studies support an inverse or positive association between coffee consumption and CRP levels among European men and women, US women, and Japanese men. Given these conflicting associations, factors such as smoking and BMI may be attributable to these variations, and the potential interaction with gender needs to be explored further. 

## Figures and Tables

**Table 1:** Participant characteristics by study *.

Study Name	Study Years	Gender	Number of Participants	Age (Years Old)	BMI	Current Smokers (%)	Alcohol	Hormone Therapy Use (%)	Coffee Consumption	Cup Conversion
DREW trial [26]	2001–2005	Women	344	57.1 ± 6.4 45–75	36.1 ± 3.9 kg/m^2^	0%	1.7 ± 2.4 g/day	46.5%	55.2% consumed < 1 cup/day	No data (used 237 mL)
NHS [18]	1976	Women	15,551	57.3	25.5 kg/m^2^	13.4%	6 g/day	41.3%	28.9% consumed ≤ 1 cup/day	1 cup = 237 mL
HPFS [18]	1986	Men	7397	62.4	25.7 kg/m^2^	6.2%	11.8 g/day	-	36.1% consumed ≤ 1 cup/day	1 cup = 237 mL
EPIC Study [22]	1992–2000	Women	10,520	51.7 (45.3–58.4)	24.2 kg/m^2^	17%	4.2 g/day	25.9%	300 mL/day	1 cup = 237 mL
		Men	4280	53.3 (46.6–59.6)	26.3 kg/m^2^	30%	14.5 g/day	-	380 mL/day	
Kyushu University Cohort Study [25]	2004–2007	Women	5918	62 49–76	22.5 kg/m^2^	6%	27.1%	No data	Median: 2 cups/day	No data (used 150 mL)
		Men	4407		23.5 kg/m^2^	32.4%	72.7%			
ATTICA study [17]	2001–2002	Women	1528	45.5 ± 13 18–87	15.6%	45.5%	No data	No data	24% consumed < 1 cup/day	1 cup = 150 mL
		Men	1514		19.7%	62.4%			9% consumed < 1 cup/day	
Singapore Prospective Study [19]	2003–2007	Both	4139	48.8 ± 11.3	23.2 kg/m^2^	11.7%	18.6%	No data	40% consumed < 1 cup/day	1 cup = 215 mL
Aichi Workers’ Cohort Study [20]	2002	Both	3317	47.6 ± 7.1 35–69	22.8 ± 2.7 kg/m^2^	29.6%	14.2 g/day	No data	Median: 1 cup/day	1 cup = 150 mL
United Kingdom Study [21]	2003–2004	Men	61	32.7	25.3 kg/m^2^	0%	14.7 g/day	No data	Mean: 1.1 cup/day	1 cup = 237 mL
FinnDiane Study [23]	1997	Both	1040	46.7 ± 0.4	25.6 kg/m^2^	13.3%	2.7 g/day	No data	12.9% consumed < 1 cup/day Mean: 3.8 ± 2.8 cups/day	1 cup = 150 mL
BELSTRESS [24]	1994–1998	Men	1031	49.0	27.0 kg/m^2^	34.9%	27.3 g/day	-	16% consumed < 1 cup/day	1 cup = 150 mL

* Mean or mean ± standard deviation; range; and/or median (25th–75th percentiles) are provided for age; mean, median, or percentage of obesity is provided for BMI; mean or percentage of alcohol consumers is provided for alcohol consumption; percentage of hormone therapy users among postmenopausal women is provided for hormone therapy use; mean, median, and/or percentage of those who consumed specific numbers of cups per day are provided for coffee consumption. Study years/calendar year(s) when the study was conducted were obtained as follows when not mentioned in the article: (1) extracting the years when participants enrolled in the study or provided information through questionnaires and other data collection procedures for cross-sectional study (ATTICA study [17]) or analysis of intervention trials or prospective studies (DREW [26], EPIC [22], Kyushu University cohort study [25], Singapore Prospective study [19], and Aichi Workers’ study [20]); (2) extracting from another article of the same study (UK study [40] and the FinnDiane study [41,42] and BELSTRESS study [43]); or (3) extracting years when blood draw and coffee intake assessments through a food frequency questionnaire (FFQ) (NHS and HPFS [18]). Lifestyle factors and median (25th–75th percentiles) age by gender for EPIC were taken from other EPIC study publications [44,45], and lifestyle factors for BELSTRESS study were taken from another BESLTRESS study publication [46].

**Table 2:** C-reactive protein levels by coffee consumption by study.

Study Name	Gender	Categories in the Original Study: Median or Mid-Point Volume (mL)					*P*-Trend *
		None	Low	Medium	High	Very High	
		Number of Participants					
		Geometric Mean c-Reactive Protein (95% Confidence Intervals) (mg/L)					
DREW trial [26]		None: 0 mL	1 cup/month to 6 cups/week: 104 mL	1 cup/day to 13 cups/week: 339 mL	≥2 cups/day: 533 mL	—	
	Women	104	86	89	65	—	0.05
		4.1 (3.4, 4.9)	4.1 (3.3, 5.0)	3.1 (2.6, 3.8)	3 (2.4, 3.8)	—	
		None: 0 mL	<1 cup/day: 119 mL	2–3 cups/day: 356 mL	≥4 cups/day: 1037 mL	—	
NHS [18]	Women	3433	4178	5653	2287	—	0.02
		4.18 (3.55, 4.92)	4.04 (3.60, 4.52)	3.25 (3.03, 3.48)	2.37 (2.16, 2.59)	—	
HPFS [18]	Men	1723	2341	2354	979	—	0.37
		1.09 (0.92, 1.29)	1.07 (0.95, 1.21)	0.97 (0.88, 1.06)	1.00 (0.88, 1.12)	—	
EPIC study [22]		None: 0 mL	Low: 103 mL	Medium–low: 297 mL	Medium–high: 451 mL	High: 745 mL	
	Men	212	1078	977	1078	935	0.01
		1.15 (1.13, 1.16)	1.16 (1.15, 1.18)	1.18 (1.17, 1.20)	1.21 (1.19, 1.22)	1.34 (1.33, 1.36)	
	Women	832	2730	2171	2730	2057	0.002
		1.42 (1.40, 1.45)	1.39 (1.36, 1.41)	1.28 (1.26, 1.30)	1.26 (1.24, 1.30)	1.16 (1.14, 1.17)	
Kyushu University Cohort Study [25]		None: 0 mL	<1 cup/day: 75 mL	1–3 cups/day: 300 mL	4–6 cups/day: 750 mL	≥7 cups/day: 1305 mL	
	Men	721	1145	1986	469	86	0.03
		0.55 (0.51, 0.59)	0.53 (0.50, 0.56)	0.51 (0.49, 0.53)	0.5 (0.46, 0.55)	0.44 (0.35, 0.55)	
	Women	892	1578	2944	444	60	0.50
		0.40 (0.38, 0.43)	0.40 (0.38, 0.42)	0.39 (0.38, 0.41)	0.41 (0.37, 0.45)	0.31 (0.24, 0.39)	
ATTICA study [17]		None: 0 mL	<200 mL/day: 100 mL	200–400 mL/day: 300 mL	>400 mL/day: 650 mL	—	
	Men	133	758	521	27	—	0.11
		2.3 (0.76, 3.84)	2.2 (0.76, 3.64)	2.9 (−0.56, 6.36)	3.1 (1.85, 4.35)	—	
	Women	366	922	211	19	—	0.21
		2.1 (0.66, 3.54)	2.0 (0.56, 3.44)	2.7 (−0.76, 6.16)	2.9 (1.36, 4.44)	—	
Singapore Prospective Study [19]		Never/rarely: 0 mL	<1 cup/day: 123 mL	1–2 cups/day: 323 mL	≥3 cups/day: 860 mL	—	
	Both	1202	475	2118	344	—	0.37
		1.31 (1.16, 1.49)	1.43 (1.25, 1.65)	1.28 (1.14, 1.44)	1.23 (1.05, 1.44)	—	
Aichi Workers’ Cohort Study [20]		<1 cup: 75 mL	1 cup: 150 mL	2–3 cups: 375 mL	≥4 cups/day: 750 mL	—	
	Both	949	803	1336	229	—	0.51
		0.43 (0.41, 0.47)	0.4 (0.37, 0.43)	0.37 (0.35, 0.40)	0.42 (0.36, 0.48)	—	
United Kingdom study [21]		—	<1 cup/day: 45 mL	1–2 cups/day: 195 mL	>2 cups/day: 435 mL	—	
	Men	—	16	20	41	—	0.94
		—	0.97 (0.77, 1.17)	0.83 (0.64, 1.02)	0.94 (0.82, 1.06)	—	
FinnDiane Study [23]		<1 cup/day: 75 mL	≥ 1 cup/day < 3: 300 mL	≥ 3 cups/day < 5: 600 mL	≥5 cups/day: 1013 mL	—	
	Both	134	230	371	305	—	0.27
		1.93 (1.56, 2.30)	1.88 (1.61, 2.16)	1.62 (1.41, 1.82)	1.68 (1.44, 1.91)	—	
BELSTRESS [24]		None	1–3 cups/day: 300 mL	>3cups/day: 750 mL	—	—	
	Men	168	415	448	—	—	0.30
		0.89 (0.75, 1.04)	0.95 (0.81, 1.11)	0.97 (0.82, 1.14)	—	—	

* *P*-trend was re-calculated based on the estimated mid-point volume (in mL) of coffee consumption in each category and obtained by treating coffee volume as continuous in a model testing a linear association between coffee consumption and CRP level.

## Supplementary Materials

The following are available online at https://www.mdpi.com/2072-6643/12/5/1349/s1, Table S1: Risk of Bias Assessment.

## Abbreviations

BMI	body mass index
CI	confidence intervals
CRP	c-reactive protein
CVD	cardiovascular disease
DREW	Dose-Response to Exercise in Women
EPIC	European Prospective Investigation into Cancer and Nutrition
FFQ	food frequency questionnaire
FinnDiane	Finnish Diabetic Nephropathy
HPFS	Health Professional Follow-Up Study
NHS	Nurses’ Health Study
UK	United Kingdom
US	United States
