# Supplementary material for: Coffee Consumption and C-Reactive Protein Levels: A Systematic Review and Meta-Analysis
Source: Nutrients. 2020 May 8;12(5):1349. doi: 10.3390/nu12051349 (PMC7285227; doi:10.3390/nu12051349)
Supplement: Supplementary file 1 [file nutrients-12-01349-s001.pdf]

Nutrients **2020**, *12*, x; doi: FOR PEER REVIEW www.mdpi.com/journal/nutrients

Supplementary Table S1: Risk of Bias Assessment*

First Author, Publication Year, Study Name	Selection	Comparability	Outcome	Total
Aresnault, 2009, Drew trial [26]	*	**	***	6
Hang, 2019, NHS/HPFS [18]	***	**	***	8
Gunter, 2017, EPIC study [22]	***	**	**	7
Maki, 2010, Kyushu University Cohort [25]	**	**	***	7
Zampelas, 2004, ATTICA study [17]	*****	**	***	10
Rebello, 2011, Singapore Prospective study [19]	***	**	***	8
Yamashita, 2012, Aichi Workers' cohort [20]	**	**	***	7
Hamer, 2006, United Kingdom study [21]	**	**	***	7
Stutz, 2018, FinnDiane study [23]	**	**	***	7
De Bacquer, 2006, BELSTRRESS study [24]	***	none	***	6

*Maximum number of stars is 5 for Selection, 2 for Comparability, and 3 for Outcome.
